# Wound-Induced Temporal Reprogramming of Gene Expression during Agarwood Formation in *Aquilaria sinensis*

**DOI:** 10.3390/plants12162901

**Published:** 2023-08-09

**Authors:** Jieru Xu, Ruyue Du, Yue Wang, Jinhui Chen

**Affiliations:** 1 Sanya Nanfan Research Institute of Hainan University, Hainan Yazhou Bay Seed Laboratory, Hainan University, Sanya 572019, China; jieruxu@hainanu.edu.cn (J.X.); ruyuedu@hainanu.edu.cn (R.D.); yuewang@hainanu.edu.cn (Y.W.); 2 Key Laboratory of Genetics and Germplasm Innovation of Tropical Special Forest Trees and Ornamental Plants, Ministry of Education/Engineering Research Center of Rare and Precious Tree Species in Hainan Province, School of Tropical Agriculture and Forestry, Hainan University, Haikou 570228, China

**Keywords:** *Aquilaria sinensis*, transcriptional regulation, mechanical wounding, lignin biosynthesis, sesquiterpene biosynthesis

## Abstract

Agarwood is a resinous heartwood of *Aquilaria sinensis* that is formed in response to mechanical wounding. However, the transcriptional response of *A. sinensis* to mechanical wounding during the agarwood formation process is still unclear. Here, three five-year-old *A. sinensis* trees were mechanically damaged by a chisel, and time-series transcriptomic analysis of xylem tissues in the treated area (TA) was performed at 15 (TA1), 70 (TA2) and 180 days after treatment (TA3). Samples from untreated areas at the corresponding time points (UA1, UA2, UA3, respectively) were collected as controls. A total of 1862 (TA1 vs. UA1), 961 (TA2 vs. UA2), 1370 (TA3 vs. UA3), 3305 (TA2 vs. TA1), 2625 (TA3 vs. TA1), 2899 (TA3 vs. TA2), 782 (UA2 vs. UA1), 4443 (UA3 vs. UA1) and 4031 (UA3 vs. UA2) genes were differentially expressed (DEGs). Functional enrichment analysis showed that DEGs were significantly enriched for secondary metabolic processes, signal transduction and transcriptional regulation processes. Most of the genes involved in lignin biosynthesis were more abundant in the TA groups, which included *phenylalanine ammonia-lyase*, *4-coumarate CoA ligase*, *cinnamate 4-hydroxylase*, *caffeoyl-CoA O-methyltransferase* and *cinnamoyl-CoA reductase*. DEGs involved in sesquiterpene biosynthesis were also identified. *Hydroxymethylglutaryl-CoA synthase*, *3-hydroxy-3-methylglutaryl-coenzyme A reductase*, *phosphomevalonate kinase* and *terpene synthase* genes were significantly increased in the TA groups, promoting sesquiterpene biosynthesis in the wounded xylem tissues. The TF-gene transcriptomic networks suggested that MYB DNA-binding, NAM, WRKY, HLH and AP2 TFs co-expressed with genes related to lignin and sesquiterpene synthesis, indicating their critical regulatory roles in the biosynthesis of these compounds. Overall, our study reveals a dynamic transcriptional response of *A. sinensis* to mechanical wounding, provides a resource for identifying candidate genes for molecular breeding of agarwood quality, and sheds light on the molecular mechanisms of agarwood formation in *A. sinensis*.

## 1. Introduction

Mechanical wounding is a common abiotic stress in plants and wounded tissues that activates specific genes and metabolic pathways and ultimately leads to the production of important secondary metabolites [1]. Agarwood is a resin wood produced by *Aquilaria* plants in response to injuries, diseases, insect attacks or decay [2,3]. The microscopic structures of the wounded *Aquilaria* wood can be classified into four zones on the basis of color and relative position: decayed, agarwood, transition and white-timber zones [4,5]. The agarwood formation process takes place in transition and white-timber zones. These two zones include the living parenchyma cells and convert starch grains into agarwood substances, ultimately leading to increased accumulation of resin in the agarwood zone [5]. These substances are found in the nearby phloem, parenchyma cells and vessels in the xylem region.

Agarwood is widely used in medicine, religious activities and in spices in China, Japan, India and other Southeast Asian countries [3]. The formation of superior agarwood takes more than 30 years, and its price is much higher than gold [3]. The major chemical components of agarwood are sesquiterpenes, 2-(2-phenethyl)chromones and flavonoids [6]. Around 300 substances have been isolated from agarwood, and more than half of them are new compounds [6]. Due to huge commercial profits and illegal trade, wild resources of *Aquilaria* plants have gradually been depleted, and thus, they are listed as an endangered species [7].

*A. sinensis* is one of the most economically important agarwood-producing species and molecularly alters the secondary metabolism in response to various stresses, leading to the accumulation of sesquiterpenoids and chromone substances [8]. Therefore, understanding their biosynthesis and regulation is crucial for studying the mechanism of agarwood formation in *A. sinensis*. While terpenoid metabolic pathways have been widely studied, little is known regarding the biosynthesis of chromone derivatives. Sesquiterpenes are synthesized by multiple enzymes via the mevalonic acid (MVA) pathway in the cytoplasm and the methylerythritol 4-phosphate (MEP) pathway in the plastids [9]. Sesquiterpene synthase is a key enzyme that converts farnesyl diphosphate (FPP) to sesquiterpenes [7]. AaWRKY1 TF activates the expression of the *amorpha-4,11-diene synthase* (*ADS*) gene by binding to the W-box of the *ADS* gene’s promoter; this finally leads to the biosynthesis of artemisinin (a sesquiterpene lactone endoperoxide) [10]. In *Arabidopsis*, MYC2 TF can interact with the DELLA protein to positively regulate the expression of sesquiterpene synthase genes (*TPS21* and *TPS11*) [11]. Treatment with methyl jasmonate (MeJA) induces the expression of sesquiterpene synthase genes (*AcC2*, *AcC3* and *AcC4*) and promotes the production of sesquiterpene *δ*-guaiene [12]. Further, Xu et al. (2013) found that mechanical wounding could induce the expression of key synthase genes, such as *3-hydroxy-3-methylglutaryl-coenzyme A reductase* (*HMGR)*, *1-deoxy-D-xylulose-5-phosphate synthase* (*DXS*), *farnesyl diphosphate synthase* (*FPPS*) and *sesquiterpene synthase* (*ASS*), as well as some genes encoding for AP2, WRKY and MYC TFs [7].

Additionally, environmental factors, such as mechanical wounding, stimulate the expression of genes and transcription factors (TFs) in plants, which in turn mount relevant defense responses. For instance, phenylpropanoid metabolism is an important secondary metabolic pathway whose genes are induced by mechanical wounding to synthesize compounds such as flavonoids and lignin, and their intermediates [13,14]. Previous studies have shown that mechanical wounding could induce the expression of *chalcone synthase* (*CHS*), *phenylalanine ammonia-lyase* (*PAL*), *cinnamate 4-hydroxylase* (*C4H*) and *4-coumarate CoA ligase* (*4CL*) genes that are associated with flavonoid biosynthesis pathways [15,16]. Similarly, rejuvenation by trunk truncation markedly promoted the accumulation of flavonol glycosides in rejuvenated *Ginkgo biloba*, and the up-regulation of genes involved in flavonoid biosynthesis was observed, including *CHS*, *flavonol synthase*, *flavonoid 3′-hydroxylase* and *dihydroflavonol 4-reductase* [17]. Wounded xylem tissues of Scots pine are known to accumulate large amounts of stilbene and lignans [18]. MYB, WRKY and AP2 TF families could play a critical role in this process by regulating key genes that are involved in phenylpropanoid biosynthesis [19].

Furthermore, there are complex signaling networks in plants that respond to wounding, which includes a variety of signaling molecules and different kinds of species-specific genes. Several hormones, such as JA, abscisic acid (ABA) and ethylene are involved in signal transduction pathways and activate defense-related genes, which accumulate in wounded tissues [20,21]. While there is a general understanding of this very complex signal transduction process during mechanical wounding, the dynamic changes in the signaling molecules and their related genes during the agarwood formation process are poorly studied. We hypothesize that understanding the signal transduction network during mechanical wounding in *A. sinensis* would provide valuable clues for elucidating the molecular mechanism of agarwood formation.

The agarwood formation process is split into three post-wounding phases, which are marked as an early (hours to 14 days), middle (>4 weeks) and late (>6 months) phase, detected by the change in the color of wounded wood, from yellow to brown and finally to black, respectively [22]. Meanwhile, the odor and chemical contents of agarwood also continue to significantly increase with time [22,23]. However, the molecular response to mechanical wounding that drives the dynamic process of agarwood accumulation in *A. sinensis* has seldom been studied. Therefore, we have attempted to fill this important gap in this investigation.

Here, three five-year-old *A. sinensis* trees were mechanically damaged to induce agarwood formation. We applied time-series transcriptomic analysis of xylem tissues in treated (TA) and untreated areas (UA) at 15, 70 and 180 days after treatment. The wound-induced genes related to secondary metabolic pathways, signal transduction and transcription regulation, which were identified with the help of high-throughput RNA-seq. The regulatory networks, involving the TFs and secondary metabolite-related genes, were constructed by co-expression analysis. The obtained results provide insights into the molecular mechanism of agarwood formation in *A. sinensis* and improve strategies to use the gene information in genetic engineering.

## 2. Results

### 2.1. Overview of RNA-seq Data Analysis

Illumina RNA-seq technology was used to sequence transcriptomes of xylem tissues that are associated with the agarwood formation process in *A. sinensis* trees. In total, six samples (UA1, UA2, UA3, TA1, TA2 and TA3) per tree for three trees (biological replicates) were collected for RNA-seq. After removing sequencing adapters and low-quality reads, we obtained 44,037,249–46,942,907 clean reads (Figure S1). These clean reads were mapped to the *A. siensis* genome, with an average mapping ratio of 91.32% (Figure S1).

### 2.2. Analysis of the Differentially Expressed Genes (DEGs)

After mechanical wounding, a total of 1862 (TA1 vs. UA1), 961 (TA2 vs. UA2) and 1370 (TA3 vs. UA3) DEGs were identified (Figure S2). When we compared the expression of genes at time-points to each other in combinations of TA2 vs. TA1, TA3 vs. TA1 and TA3 vs. TA2, we obtained 3305, 2625, and 2899 DEGs, respectively (Figure S2). Among the untreated groups, we found 782, 4443 and 4031 DEGs in UA2 vs. UA1, UA3 vs. UA1 and UA3 vs. UA2 comparisons, respectively (Figure S2). We found that the majority of DEGs in treated areas compared with their controls were up-regulated (Figure S2). Among the treated groups, the number of up-regulated genes in TA3 vs. TA2, TA2 vs. TA1 and TA3 vs. TA1 was not different, while the number of down-regulated genes was at a maximum in TA2 vs. TA1 (Figure S2).

### 2.3. Enrichment Analysis of DEGs

GO enrichment analysis was performed to identify the biological functions of DEGs at a significance level of *p*_adj_ < 0.05. DEGs were significantly assigned to the three categories of biological processes, cellular components and molecular functions. GO terms related to biosynthesis of the secondary metabolic process, oxidoreductase activity, response to wounding, response to bacterium, active transmembrane transporter activity and monooxygenase activity were enriched (Figure S3). To better understand their function, DEGs were mapped to the KEGG database. Here, significant enrichment was notably related to flavonoid biosynthesis, phenylpropanoid biosynthesis, glutathione metabolism and plant–pathogen interaction pathways (Figure S4).

### 2.4. Identification and Characterization of Transcription Factors (TFs) in Response to Mechanical Wounding

**Figure 1:**
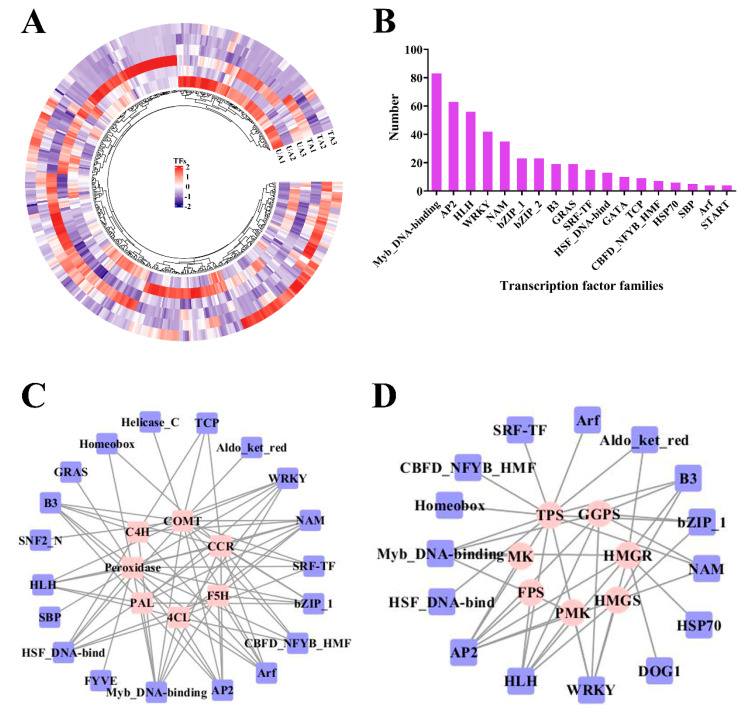
Differentially expressed transcription factors (TFs) in response to mechanical wounding in *Aquilaria sinensis*. (**A**) Heatmap of DEGs encoding for TFs. FPKM values of the individual genes were normalized across the rows and the heatmap was visualized in R. (**B**) Bar chart of major TF families of these TFs. (**C**) The co−expression network of TFs and DEGs involved in lignin biosynthesis. (**D**) The co−expression network of TFs and DEGs involved in sesquiterpene biosynthesis. The genes involved in secondary metabolism are represented by pink circles and transcription factors are represented by blue rectangles.

In this work, we detected 451 DEGs that correspond to TFs (Figure 1A). Most of the genes belonged to MYB DNA-binding (83), AP2 (63), HLH (56), WRKY (42), NAM (35) and bZIP-1 (23) families (Figure 1B). In the TA1 vs. UT1 comparison, 128 TF-encoding DEGs were identified, of which 86 were up-regulated and 42 were down-regulated (Table S2). In TA2 vs. UA2, 108 TF-encoding DEGs were detected, of which 98 were up-regulated and 10 were down-regulated (Table S2). In TA3 vs. UA3, 126 TF-encoding DEGs were annotated, including WRKY, AP2, HLH and NAM TF families (Table S2). Moreover, we identified many significantly up-regulated TF genes in TA1 vs. UA1, TA2 vs. UA2 and TA3 vs. UA3, and the majority belonged to MYB DNA-binding, NAM, AP2, HLH and WRKY TF families (Table S2).

### 2.5. Differential Expression of Genes Involved in Lignin Biosynthesis Pathway

**Figure 2:**
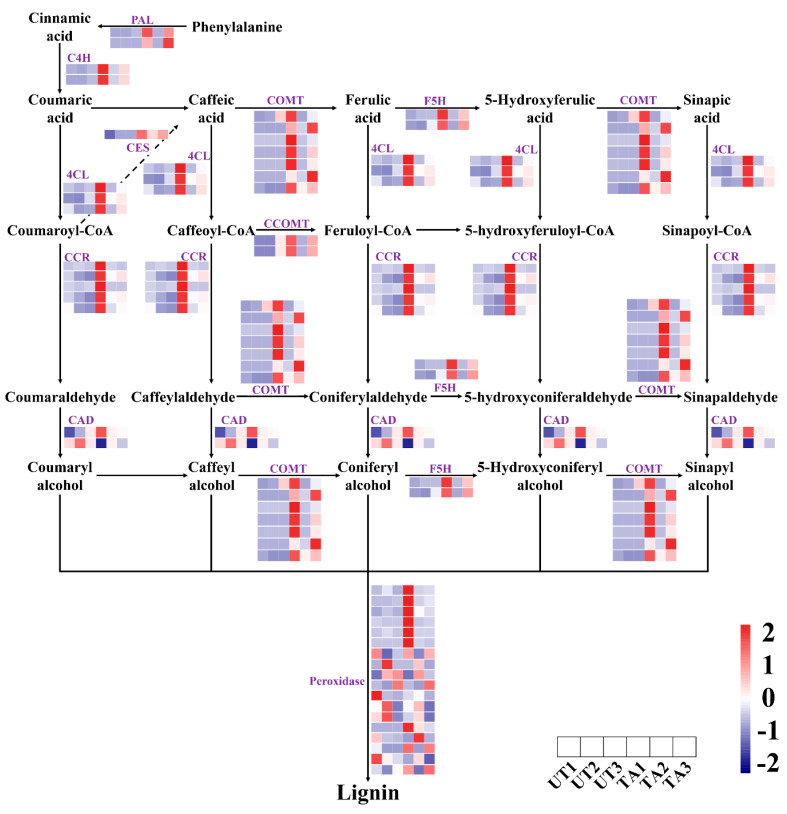
Expression analysis of genes involved in the phenylpropanoid biosynthesis pathway during response to mechanical wounding in *Aquilaria sinensis*. FPKM values of the individual genes were normalized across the rows and the heatmap was visualized in R. Each row represents the normalized gene expression level of individual gene and each column represents the expression level from different samples (in order represented in the horizontal scale). PAL, phenylalanine ammonia−lyase; C4H, cinnamate 4−hydroxylase; 4CL, 4−coumarate CoA ligase; CCR, cinnamoyl−CoA reductase; COMT, caffeic acid O−methyltransferase; CAD, cinnamyl alcohol dehydrogenase; F5H, ferulate 5−hydroxylase; CSE, caffeoylshikimate esterase; CCoAOMT, caffeoyl−CoA O−methyltransferase.

We found 44 DEGs that were involved in lignin biosynthesis, including PAL, 4CL, C4H, cinnamyl alcohol dehydrogenase (CAD), cinnamoyl-CoA reductase (CCR), caffeic acid O-methyltransferase (COMT), caffeoyl-CoA O-methyltransferase (CCoAMT) and pexoridase-encoding genes (Figure 2). Among them, a total of 30, 6 and 6 DEGs were detected in TA1 vs. UA1, TA2 vs. UA2 and TA3 vs. UA3, respectively. Moreover, PAL, 4CL, C4H, caffeoylshikimate esterase (CSE), ferulate 5-hydroxylase (F5H), CCoAMT, CCR and COMT genes, as well as several pexoridase-encoding genes were up-regulated in the TA groups as compared to their respective (untreated) controls (Figure 2), indicating their contribution towards the biosynthesis of lignin. The expression of CCoAMT, CCR, 4CL, COMT, F5H, CSE, CAD and C4H genes were highest in TA1, followed by TA3 and TA2 (Figure 2). At the same time, the expression levels of most of the genes involved in lignin biosynthesis had little differences across UA1, UA2 and UA3 (Figure 2).

To detect the regulatory factors of DEGs that are involved in lignin biosynthesis, a co-expression network was constructed with the help of Pearson’s correlation coefficients (Figure 1C). The TF families that were associated with these genes included MYB DNA-binding, AP2, NAM, WRKY, HLH, bZIP_1, HSF DNA-bind, B3, GRAS, ARF, TCP and GATA TFs (Figure 1C). Here, *PAL* genes were mainly co-expressed with AP2, HLH, NAM and WRKY families (Figure 1C). Similarly, the *4CL* genes were co-expressed with ARF, HLH, B3, MYB DNA-binding and WRKY families (Figure 1C).

### 2.6. Differential Expression of Genes Involved in Sesquiterpene Biosynthesis Pathway

**Figure 3:**
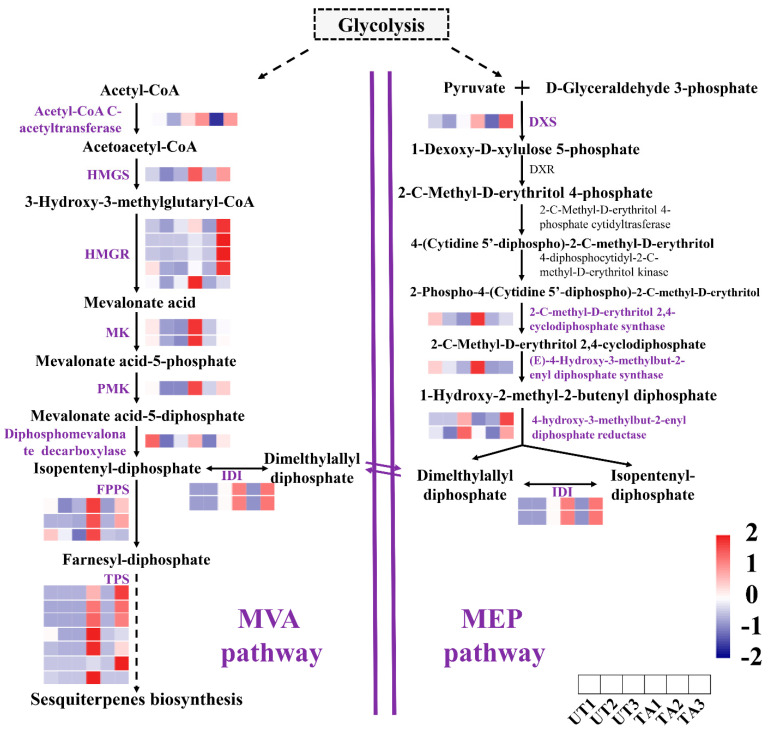
Analysis of expression of genes involved in the sesquiterpene biosynthetic pathway in *Aquilaria sinensis* in response to mechanical wounding. FPKM values of the individual genes were normalized across the rows and the heatmap was visualized in R. Each row represents the normalized gene expression level and each column represents different conditions (in order represented in the horizontal scale). HMGS: hydroxymethylglutaryl−CoA synthase; HMGR: 3-hydroxy-3-methylglutaryl-coenzyme A reductase; MK: melalonate kinase; PMK: phosphomevalonate kinase; IDI: isopentyl-diphosphate Delta-isomerase; FPPS: farnesyl diphosphate synthase; TPS: terpene synthase; DXS: 1-deoxy-D-xylulose-5-phosphate synthase.

A total of 37 DEGs were significantly enriched in the terpenoid backbone biosynthesis and the sesquiterpene biosynthesis pathways (*p*_adj_ < 0.05). In sesquiterpene biosynthesis, a total of 10, 3 and 10 up-regulated genes were detected in TA1 vs. UA1, TA2 vs. UA2 and TA3 vs. UA3 comparisons, respectively. Among them, the expression of *HMGS*, *HMGR* and *PMK* was significantly increased in all the TA groups. For example, the *HMGR* genes were up-regulated by more than six-fold in TA3 as compared to UA3 (Figure 3). Further, the expressions of seven genes encoding for terpene synthase (TPS) were significantly increased by about 6- to 16-fold in TA groups (Figure 3). The majority of sesquiterpene biosynthesis genes were expressed at the highest levels in TA3, followed by TA1 and TA2 (TA3 > TA1 > TA2; Figure 3). At the same time, expression of the majority of these genes was not significantly different in the UA groups (Figure 3).

Co-expression of TFs and genes involved in the sesquiterpene biosynthesis pathway was analyzed by Pearson’s correlation, their differential expression patterns were analyzed and a correlation network was constructed (Figure 1D). The *TPS* genes were mainly co-expressed with AP2, HLH, MYB DNA-binding, WRKY and NAM TFs (Figure 1D). The *HMGR* genes were mainly co-expressed with WRKY, AP2 and HLH TFs (Figure 1D). Similarly, *PMK* was co-expressed with AP2, WRKY, MYB DNA-binding and HLH TFs (Figure 1D). Further, we found that HLH, AP2 and WRKY TFs could be co-expressed with the majority of sesquiterpene synthetic genes (Figure 1D), indicating their critical roles in the biosynthesis of sesquiterpenes.

### 2.7. Differential Expression of Genes Involved in Hormone Signal Transduction

**Figure 4:**
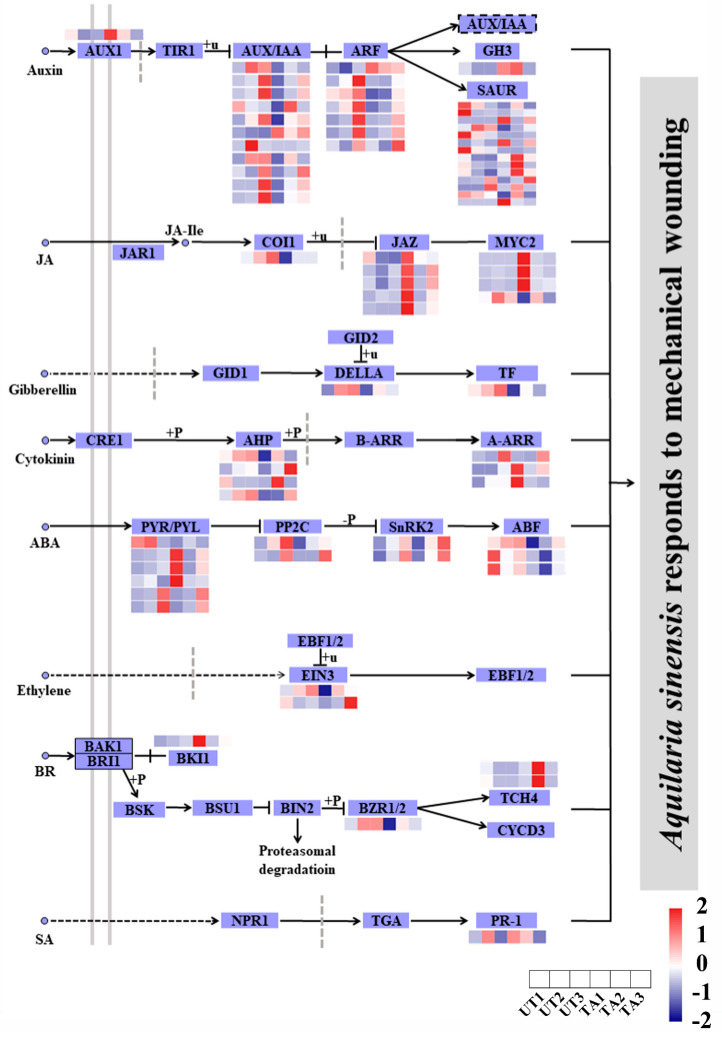
Expression analysis of genes in plant hormone signal transduction pathways in *Aquilaria sinensis* in response to mechanical wounding. FPKM values of the individual genes were normalized across the rows and the heatmap was visualized in R. Each row represents the normalized gene expression level, whereas columns represent different conditions.

A total of 56 DEGs were annotated to be involved in plant hormone signal transduction (*p*_adj_ < 0.05), of which most were from auxin, followed by JA, ABA and ethylene pathways (Figure 4). Most of these genes were up-regulated after wounding in the three comparisons, TA1 vs. UA1, TA2 vs. UA2 and TA3 vs. UA3 (Figure 4). In the auxin signaling pathway, the majority of the *AUX/IAA* and *SAUR* genes were down-regulated in TA1 vs. UA1, while *auxin-induced protein 22D* (*AUX22D*) and six *SAUR* genes were up-regulated at TA2 or TA3 time points as compared with UA2 or UA3, respectively (Figure 4). Further, *ARF5* was up-regulated in TA2 vs. UA2, while *ARF9* and *ARF2* were down-regulated in this comparison group (Figure 4). In the JA signaling pathway, *JAZ* and *MYC2* genes showed an up-regulation in the TA groups, while one *MYC2* and one *COI1* gene were significantly down-regulated in TA1 compared with UA1 (Figure 4). In the ABA signaling pathway, the *ABF* genes were down-regulated in TA1 and TA2 as compared with UA1 or UA2, respectively, whereas three *PYR/PYL* genes were up-regulated in TA1 vs. UA1 (Figure 4). Several DEGs related to gibberellin, cytokinin, ethylene, brassinosteroid (BR) and salicylic acid (SA) signaling pathways were also found, such as *TCH4* (BR signaling pathway) and *PR-1* (SA signaling pathway) genes (Figure 4). These results indicate that the agarwood formation in *A. sinensis* could involve a complex hormone network. Further, we infer that mechanical wounding had little effect on hormone signal transductions in untreated xylem tissues of *A. sinensis* as most of the aforementioned genes had no significant changes in the UA groups (Figure 4).

### 2.8. RNA-seq Verification by qRT-PCR

**Figure 5:**
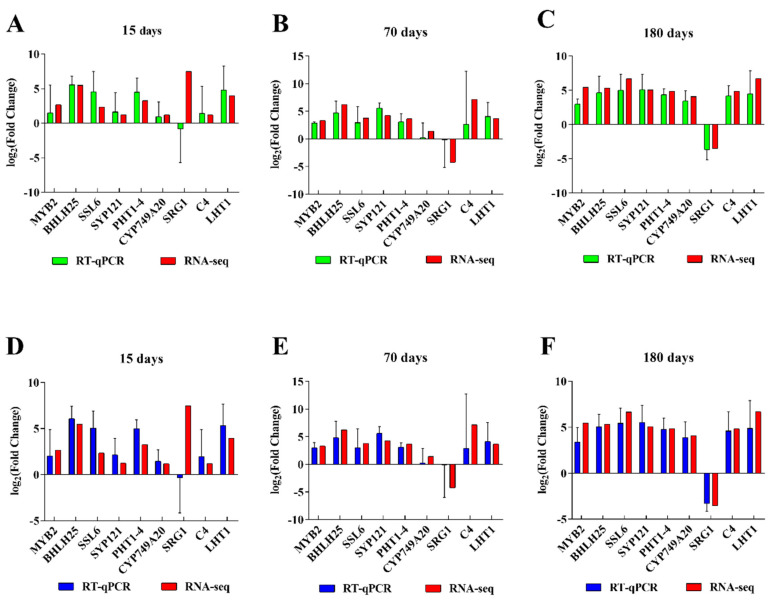
The expression levels of genes were detected by RT−qPCR. The *ubiquitin* (**A**–**C**) and *histone* (**D**–**F**) genes were used as internal controls.

To validate the accuracy and reliability of our RNA-seq data with qRT-PCR, we randomly selected nine DEGs, which were closely related to the response to mechanical wounding. Although the gene expression levels from qRT-PCR did not exactly match the RNA-seq results, similar trends across the two datasets were observed (Figure 5). These similar expression patterns demonstrate the reliability of the data obtained by high-throughput sequencing.

## 3. Discussion

Plant response to stresses involves a complex gene network, which often produces important secondary metabolites [24], such as in *Senna tora* [13], *Dendrobium officinale* [25] and *A. sinensis* [7]. Agarwood formation is one such process, where mechanical methods are often used to treat *A. sinensis* stems. As a consequence of mechanical wounding, the quality of agarwood obtained is very close to that of natural agarwood. However, there are only a few studies on transcriptomic changes that occur in response to mechanical wounding during the agarwood formation process. RNA-seq is an effective tool to accurately reveal transcriptomic information during plant defense responses [13,15,16]. Therefore, we deployed RNA-seq technology to explore time-dependent transcriptomic changes during wounding-induced agarwood formation in xylem of *A. sinensis*. Large-scale and complex changes in gene expression patterns were observed. GO and KEGG enrichment analysis of DEGs showed that they were significantly altered by secondary metabolic processes, signal transduction and transcriptional regulatory processes in response to mechanical stress (Figures S3 and S4). These results indicate that the transcriptome of *A. sinensis* was remarkably affected during a response to mechanical wounding. Moreover, the differentially regulated genes could be a resource for research on key genes associated with agarwood formation.

### 3.1. Transcription Factors during A. sinensis Response to Mechanical Wounding

Members of AP2, MYB, WRKY, bHLH, HSF and NAC TF families are known to be important regulators of abiotic stress responses [26,27,28,29,30,31]. In this study, a total of 451 TF-encoding DEGs were identified, which included MYB DNA-binding, AP2, HLH, WRKY, NAM, bZIP_1, B3, GRAS and HSF families (Figure 1A,B). Of these, we shall discuss a few important roles of TF families. For instance, WRKY is one of the most important TF families, as several of them (such as WRKY40, WRKY33, WRKY53, WRKY12 and WRKY11) are involved in the *Arabidopsis* response to mechanical wounding [32]. Here, there was a total of 42 WRKY-encoding DEGs, of which 12, 16 and 27 WRKY were up-regulated in TA1 vs. UA1, TA2 vs. UA2 and TA3 vs. UA3 (Figure 1A; Table S2), respectively, indicating that the WRKY family could play an important regulatory role in *A. sinensis* response to mechanical wounding. WRKYs are known to play important roles in regulating plant defenses against pathogens [33,34] and insects [35], as well as during abiotic stresses such as salinity and drought [36]. WRKYs modulate such responses by regulating phytohormone synthesis and response, like that of SA and JA [34]. For instance, OsWRKY6 increased SA contents by directly binding to the promoter of *Oryza sativa isochorismate synthase 1* (*OsICS1*), thus regulating plant defense responses [33,34]. *Triticum Aestivum WRKY75-A* (*TaWRKY75-A*) could function as a key stress-resistant regulator to adapt to drought and salt stress by modulating the JA biosynthetic and metabolic pathways [36]. WRKYs, along with AP2 TFs, also act as positive regulators of *TPS* genes in *Gossypium arboreum* [37], *Arabidopsis* [11] and *Artemisia annua* [10,38].

We identified 63 AP2-encoding genes, the majority of which were up-regulated in the TA groups (Table S2), indicating their critical roles in agarwood formation. AP2 TFs not only act during a plant’s growth and development but also respond to biotic and abiotic stresses and participate in immune responses [26,39]. The PsAP2 TF from *Papaver somniferum* was highly activated in response to wounding, and the *PsAP2* overexpression in tobacco enhanced tolerance towards both abiotic and biotic stress [39]. ORA47 (octadecanoid-responsive AP2/ERF-domain TF 47) plays an important role in the biosynthesis of JA and ABA when plants respond to wounding and water stress. Additionally, AP2 TFs also mediate plant stress response by involvement in the biosynthesis of secondary metabolites [40]. For example, glycoalkaloid metabolism 9 (GAME9, an AP2 TF) functions as a regulator of alkaloid production and controls the biosynthesis of steroidal glycoalkaloids [41].

Additionally, MYB TFs regulate plant secondary metabolism and hormone signal transduction processes, often in response to abiotic stresses, such as mechanical wounding, and biotic stresses such as pest attacks and diseases [31]. *RrMYB5* and *RrMYB10* were induced by wounding and oxidation; they contributed to the biosynthesis of proanthocyanidins in *Rosa rugosa* by activating the expression of flavonoid structural genes, such as *RrANR* and *RrDFR*, and thus enhanced plant tolerance to wounding and oxidation stresses [42]. Mechanical wounding induced the expression of *MYB28* in flowering plants, which supported the accumulation of aliphatic glucosinolates, thereby reducing insect performance [43]. Moreover, overexpression of *MdMYB9/11/12* was found to increase the accumulation of proanthocyanidins in tobacco and Arabidopsis [44]. We found that many MYB family members were induced in different time spans after wounding of *A. sinensis* (Table S2). It indicates that MYB TFs may play an important role in the regulation of growth and recovery responses of *A. sinensis* during mechanical wounding.

In conclusion, identification of TFs in this study is expected to provide useful information for future research on the regulatory mechanisms deployed by *A. sinensis* and other woody plants during their response to mechanical wounding.

### 3.2. Lignin Biosynthesis during A. sinensis Response to Mechanical Wounding

In response to various stresses, plants regulate their lignin contents to strengthen cell walls and avoid cell rupture [45,46]. Lignin is synthesized through the phenylpropanoid biosynthetic pathway, which involves enzymes such as PAL, 4CL, COMT, CAD, CCR and CCoAMT [45]. Most of the genes related to lignin biosynthesis were up-regulated in the TA groups as compared with their controls (Figure 2). The expression levels of *CCoAMT*, *CCR*, *4CL*, *COMT*, *F5H*, *CSE*, *CAD* and *C4H* were most abundant in TA1 (TA2 < TA3 < TA1), while the expression levels of *PAL* genes were most abundant in TA3 (TA2 < TA1 < TA3) (Figure 2). This indicates that the lignin biosynthesis process may be promoted during mechanical wounding.

Important TF families that are involved in lignin synthesis include MYB, WRKY and HLH families [47,48,49]. We found that MYB DNA-binding, AP2, NAM, WRKY, HLH and other families may regulate the key genes related to lignin biosynthesis (Figure 1C). Previous studies have shown that the overexpression of *PtoMYB74*, *PtoMYB92* and *PtoMYB216* could activate the expression of key genes related to the biosynthesis of lignin and cellulose, promote the formation of the secondary cell wall and thicken the woody cells, and finally, were involved in the regulation of wood formation [50,51,52]. Similarly, MYB58 and MYB63 could function as transcriptional regulators to specifically induce lignin biosynthetic genes during *A. thaliana* secondary wall formation [53]. The R2R3-MYB, bHLH, WDR and MBW complex (MYB-bHLH-WD40) could also regulate the expression of phenylpropanoids in plants [54,55].

In summary, MYB, HLH, WRKY, AP2 and NAM TF families might play an important regulatory role in lignin biosynthesis in *A. sinensis*.

### 3.3. Sesquiterpene Biosynthesis during A. sinensis Response to Mechanical Wounding

When *A. sinensis* is subjected to mechanical stress, it activates its immune system and produces secondary substances such as sesquiterpene and 2-(2-phenylethyl) chromone derivatives [5,23]. We attempted to explore the key genes related to the synthesis of these defense metabolites. Sesquiterpenoids are mainly synthesized through a series of reactions of the MVA pathway [9], where HMGS and HMGR have been recognized as important enzymes. Further, PMK is an important ATP-dependent enzyme that regulates the construction of the sesquiterpene backbone [9]. In this study, the expression levels of *HMGS*, *HMGR* and *PMK* were significantly increased in the TA groups as compared with the UA groups at the three timepoints. These results indicate an increased sesquiterpene synthesis in *A. sinensis* during mechanical wounding (Figure 3). Additionally, the expression of TPS genes, which are important for the synthesis of terpenes, was most abundant in the TA groups (Figure 3). This indicated that terpene synthesis may be enhanced in the mechanically damaged areas of *A. sinensis*.

Sesquiterpene biosynthesis is a complex and multistep process that is regulated by various TFs. AP2, MYC, MYB and WRKY TFs could control terpene biosynthesis in plants [9,37,56,57]. AtMYC2 directly binds to the promoters of sesquiterpene synthase genes (*TPS21* and *TPS11*) and activates their expression, thereby inducing the biosynthesis of sesquiterpenes, such as (*E*)-β-caryophyllene [11]. Further, PtMYB14 acts as a potential regulator of the MVA pathway and JA metabolism, which leads to the accumulation of volatile terpenoids in conifers [58]. At the same time, in *Citrus sinensis*, the AP2/ERF TF was positively correlated with the *CsTPS1* expression [56]. Here, we found that 15 TF families, including homeobox, MYB DNA-binding, HLH, WRKY, ARF, AP2, NAM, bZIP1, B3 and HSF DNA-bind, may play important roles in sesquiterpene synthesis by regulating its biosynthesis genes, such as *HMGR*, *PMK* and *TPS* (Figure 1D).

### 3.4. Hormone Signal Transduction during A. sinensis Response to Mechanical Wounding

Hormone contents are closely related to a plant’s growth, development and response to stresses. In response to mechanical wounding, hormones such as JA and auxin resume regulatory roles [59]. Jasmonic acid ZIM domain (JAZ) proteins are important transcriptional regulators of JA-response genes [60]. In healthy *A. sinensis*, AsMYC2 without targeting *ASS1* is recruited by the AsJAZ1 protein to form COI1-JAZ-MYC2 complexes, and thus, the expression of the *δ-guaiene synthase ASS1* is very low [61]. However, in wounded *A. sinensis*, AsMYC2 is released from the complexes and directly targets and activates *ASS1* expression, contributing to the biosynthesis of agarwood sesquiterpenes [61]. We found that three *MYC2* genes were up-regulated in TA groups and *COI1* was down-regulated in TA1 vs. UA1 (Figure 4), supporting the biosynthesis and accumulation of agarwood sesquiterpenes. On the other hand, ARFs are responsible for regulating multiple plant processes, including lateral root formation, fruit germination, apical dominance and cellular senescence [62]. When an ARF binds to auxin response AuxRE elements, it activates or represses the expression of auxin-inducible genes [62]. Aux/IAA proteins are key components of the auxin response. In *Oryza sativa*, the *AUX/IAA* genes could be induced by exogenous auxin and drought stress [63]. In *Trifolium repens*, the *IAA27* gene was down-regulated by auxin, affecting fruit growth and root development [64]. In our study, AUX- and ARF-encoding genes showed different expression profiles in the TA groups compared with the UA groups. Additionally, SAUR-encoding genes also showed different expression patterns in TA1 vs. UA1, TA2 vs. UA2 and TA3 vs. UA3 (Figure 4). SAURs induce protein phosphatases to mediate H^+^-ATPase activity [65]. The *SAUR* genes could function as positive regulators of cell expansion in *Arabidopsis*, such as *SAUR19* and *SAUR63* [66,67]. Therefore, we predict that these genes may play a role in regulating cell expansion of *A. sinensis* during mechanical wounding.

## 4. Materials and Methods

### 4.1. Sample Collection and Wounding Treatment

*A. sinensis* trees were grown in an agarwood base in Ding’an County, Hainan Province, China (19°38′48″ N, 110°15′07″ E, elevation ~66 m) to the age of five years. Three well-grown trees (about 5 m in height and 8 cm in diameter) were selected for wounding treatment to induce agarwood, which was carried out in the month of July 2020. A fan-shaped wound, with a depth of 1–4 cm, was inflicted on the stems of these three trees at a height of 1 m with the help of a chisel. The xylem tissues near the wound site were collected at 15 (TA1), 70 (TA2) and 180 days (TA3) after mechanical wounding per tree for the three trees. Meanwhile, the xylem tissues distant from the wound site (1 m above the wound) were also collected at 15 (UA1), 70 (UA2) and 180 days (UA3) after mechanical wounding as controls in the same manner from each of the three trees. The collected samples were snap frozen in liquid nitrogen and stored at −80 °C in a freezer. The experiment comprised three biological replicates for three independent trees at each timepoint.

### 4.2. RNA Extraction and Sequencing

Woody samples were grounded to powders and the xylem total RNA was extracted by using the RNAprep pure plant plus kit (Tiangen, Beijing, China). RNA quality was initially examined on a 2% agarose gel. NanoDrop and the Agilent Bioanalyzer 2100 (Agilent Technologies, Santa Clara, CA, USA) were also used to further test the RNA purity and integrity. RNA quality with A260/A230 ≥ 1.8, 1.8 ≤ A260/A280 ≤ 2.1, and RNA integrity number (RIN) > 6.8 were selected for subsequent experiments. Only high-quality RNA-seq libraries were constructed and sequenced using Illumina NovaSeq 6000 platform (Illumina, San Diego, CA, USA).

### 4.3. Transcriptome Analysis

The clean reads were obtained after removing low-quality and adapter sequences from the raw data. We downloaded the *A. sinensis* genome files (http://gigadb.org/dataset/view/id/100702; accessed on 21 January 2020), and they were used as a reference for aligning the paired-end clean reads with the help of HISAT2 (v2.0.5) [68]. The number of reads mapped onto the reference genome was calculated with the help of featureCounts (1.5.0-p3) [69], and fragments per kilobase of transcript per million fragments mapped read (FPKM) values were also obtained. Analysis of different expression levels between two conditions was performed with the help of DESeq2 (v1.20.0) [70]. Genes with |log_2_(fold change)| ≥ 1 and adjusted *p*-values (*p*_adj_) ≤ 0.05 after Benjamini and Hochberg’s correction [71] were defined as differentially expressed (DEGs). Nine pairwise comparisons were performed: TA1 vs. UA1, TA2 vs. UA2, TA3 vs. UA3, TA2 vs. TA1, TA3 vs. TA1, TA3 vs. TA2, UA2 vs. UA1, UA3 vs. UA1 and UA3 vs. UA2. GO term and KEGG enrichment analysis of DEGs were performed with the help of clusterProfiler (3.4.4) [72]. Terms with *p*_adj_ < 0.05 were considered significantly enriched.

The Pearson’s correlation coefficients between the TF DEGs and DEGs involved in lignin and sesquiterpene biosynthesis pathways were calculated by using their FPKM values in R (v 4.0.1). The coefficients between genes and TFs with |cor| > 0.9 and *p*-value < 0.001 were regarded as significant. The *p*-value was determined using the permutation test method [73]. Visualization of TF-gene co-expression network was performed with the help of Cytoscape (v 3.7.2) [74].

### 4.4. Validation of Transcriptome Data by qRT-PCR

Total RNA from 6 samples (UA1, UA2, UA3, TA1, TA2, TA3) was extracted as described above and used as a template for cDNA synthesis (GoScript™ Reverse Transcription System, Promega, Madison, WI, USA). Genes associated with *A. sinensis* responses to mechanical wounding were selected for quantitative real-time polymerase chain reaction (qRT-PCR) (Table S1). Gene-specific primers were designed with the help of Primer Premier v5 (Premier Biosoft International, Palo Alto, CA, USA). The primer design principles were as follows: PCR product length of 150–250 bp, melting temperature (T_m_ value) of 55–60 °C and GC content of 45–50%. The qRT-PCR was performed with the help of TB Green^®^Premix Ex TaqTM kit (Tli RNaseH Plus, Takara, Beijing, China). The qRT-PCR procedure was performed as follows: a cycle of 94 °C for 2 min and 40 cycles of 95 °C for 15 s and 60 °C for 30 s. We selected *histone* and *ubiquitin* as internal reference genes. The relative expression was calculated by the 2^−ΔΔCt^ method [75].

## 5. Conclusions

Our study reveals the dynamic transcriptomic changes in *A. sinensis* in response to mechanical wounding. RNA-seq was performed on xylem tissues at three time points/stages of agarwood formation. The differential gene expression analysis could retain 1862 (TA1 vs. UA1), 961 (TA2 vs. UA2), 1370 (TA3 vs. UA3), 3305 (TA2 vs. TA1), 2625 (TA3 vs. TA1), 2899 (TA3 vs. TA2), 782 (UA2 vs. UA1), 4443 (UA3 vs. UA1) and 4031 (UA3 vs. UA2) DEGs representative of the agarwood formation process. With the help of GO and KEGG analysis, we infer that sesquiterpenoid, phenylpropanoid and lignin biosynthesis pathways were up-regulated over time after wounding treatment, which could promote the biosynthesis of sesquiterpenes and lignin in *A. sinensis*. The transcriptomic networks showed that MYB DNA-binding, NAM, WRKY, HLH and AP2 TFs could be potential regulators for genes involved in lignin and sesquiterpene biosynthesis. In conclusion, this study provides insights into the dynamic transcriptional response to mechanical wounding and sheds light on the molecular mechanisms of agarwood wood formation in *A. sinensis*.

## Data Availability

The sequencing data for *A. sinensis* reported in this paper have been deposited at the Genome Sequence Archive in BIG Data Center (BIG Data Center Members, 2019), Beijing Institute of Genomics (BIG), Chinese Academy of Sciences under accession numbers CRA008994, CRA011674 and CRA006751.

## Supplementary Materials

The following supporting information can be downloaded at: https://www.mdpi.com/article/10.3390/plants12162901/s1, Figure S1: Overview of *Aquilaria sinensis* RNA-seq results; Figure S2: DEGs in *Aquilaria sinensis* xylem tissues during agarwood formation; Figure S3: The annotation of DEGs in GO terms; Figure S4: Significantly enriched KEGG pathways; Table S1: Primer pairs used in qRT-PCR assays in this study; Table S2: Differential expression of genes corresponding to transcription factors (TFs).
