# Supplementary material for: Wound-Induced Temporal Reprogramming of Gene Expression during Agarwood Formation in Aquilaria sinensis
Source: Plants (Basel). 2023 Aug 9;12(16):2901. doi: 10.3390/plants12162901 (PMC10459772; doi:10.3390/plants12162901)
Supplement: Supplementary file 1 [file plants-12-02901-s001.zip › plants-2515770-supplementary.pdf]

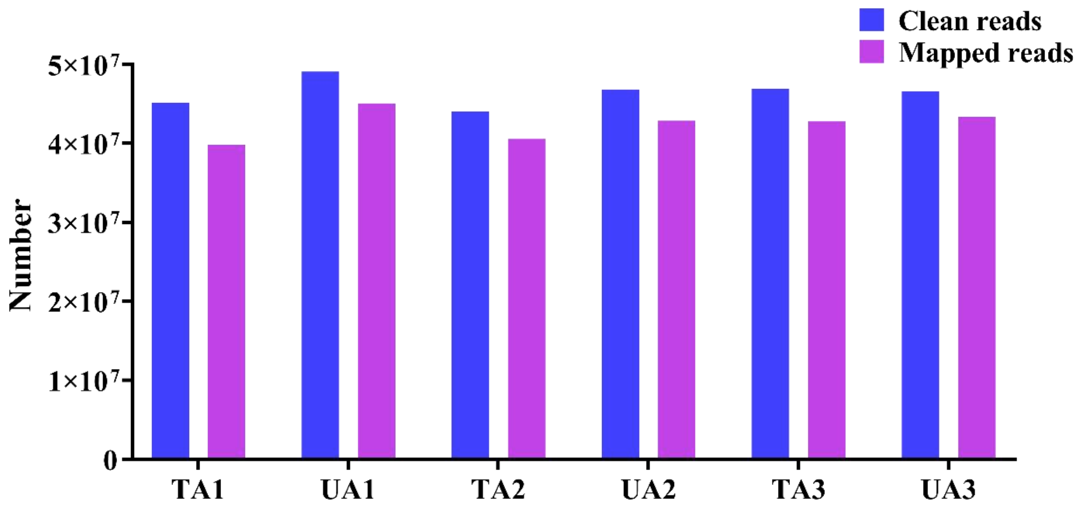

Figure S1. Overview of *Aquilaria sinensis* RNA-seq results. Numbers represent the average of three biological replicates.

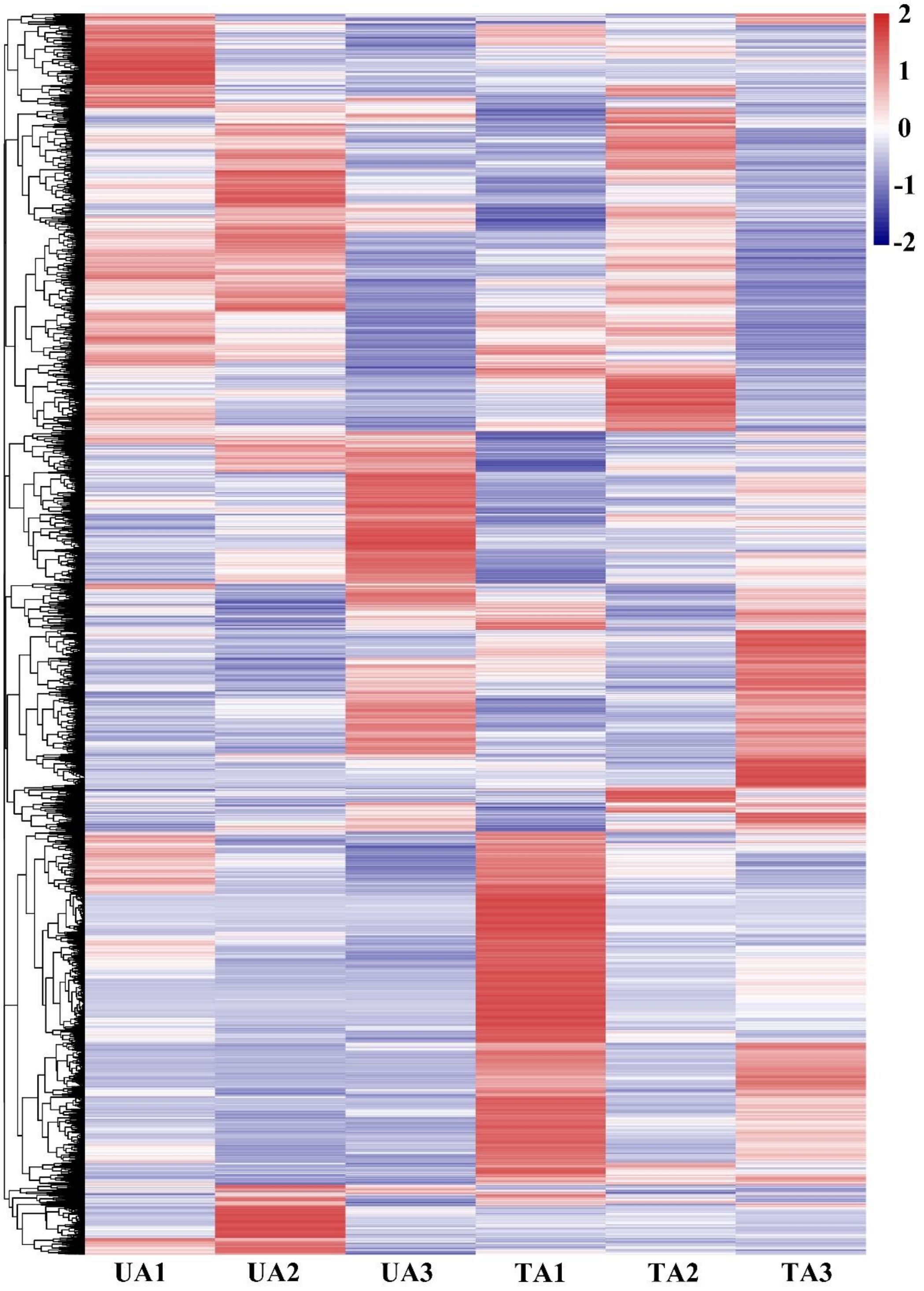

Figure S2. DEGs in *Aquilaria sinensis* xylem tissues during agarwood formation. FPKM values of the individual genes were normalized across the rows and the heatmap was visualized in R. Each rows represent the normalized gene expression level whereas columns represent different conditions.

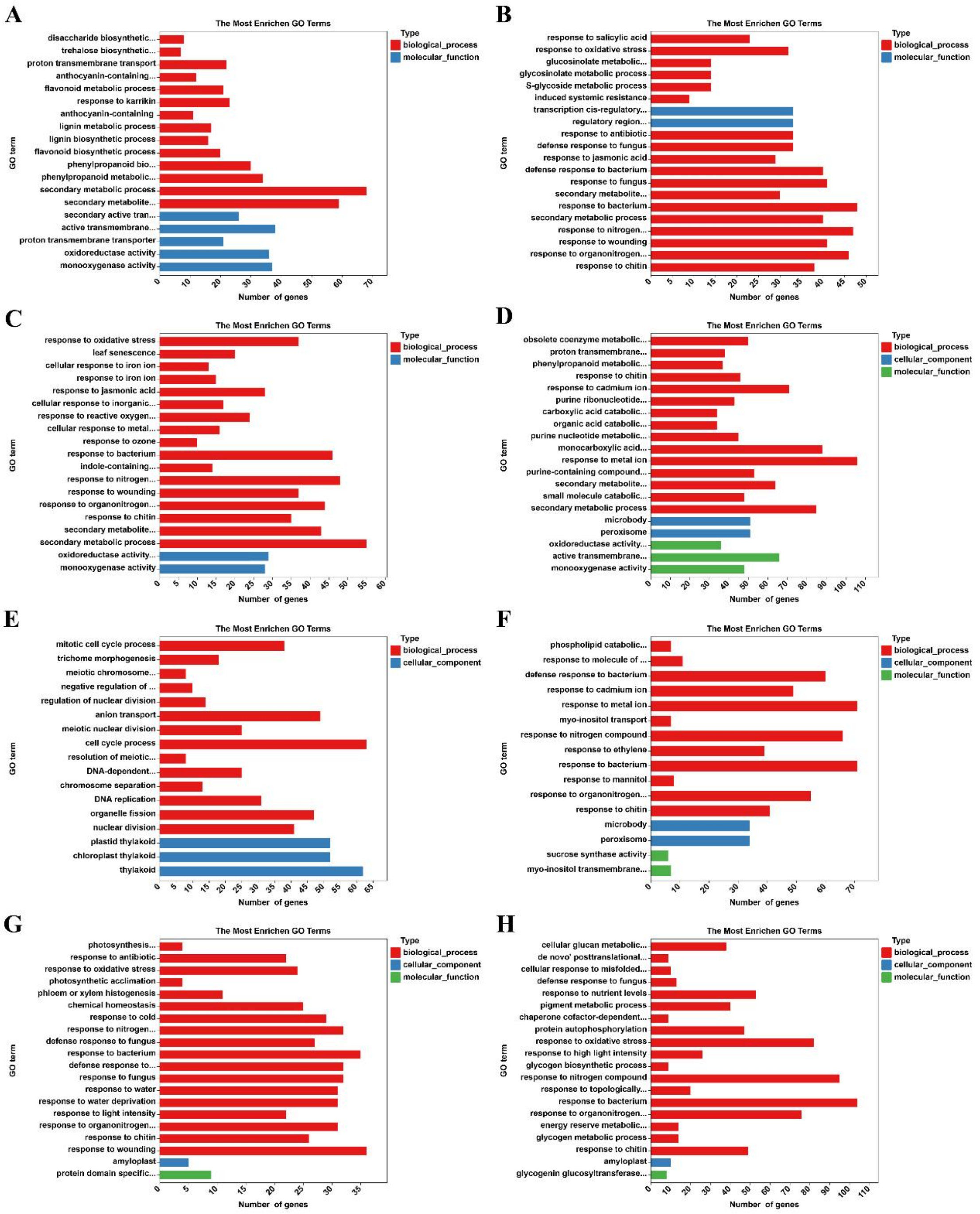

Figure S3. The annotation of DEGs in GO terms. The functions of DEGs included three GO categories: biological process, cellular component, and molecular function. The x-axis represents the number of genes, and the y-axis represents the GO term. Bar plot of GO analysis for the DEGs identified in TA1 vs. UA1 (A), TA2 vs. UA2 (B), TA3 vs. UA3 (C), TA2 vs. TA1 (D), TA3 vs. TA1 (E), TA3 vs. TA2 (F), UA2 vs. UA1 (G), and UA3 vs. UA1 (H), respectively.

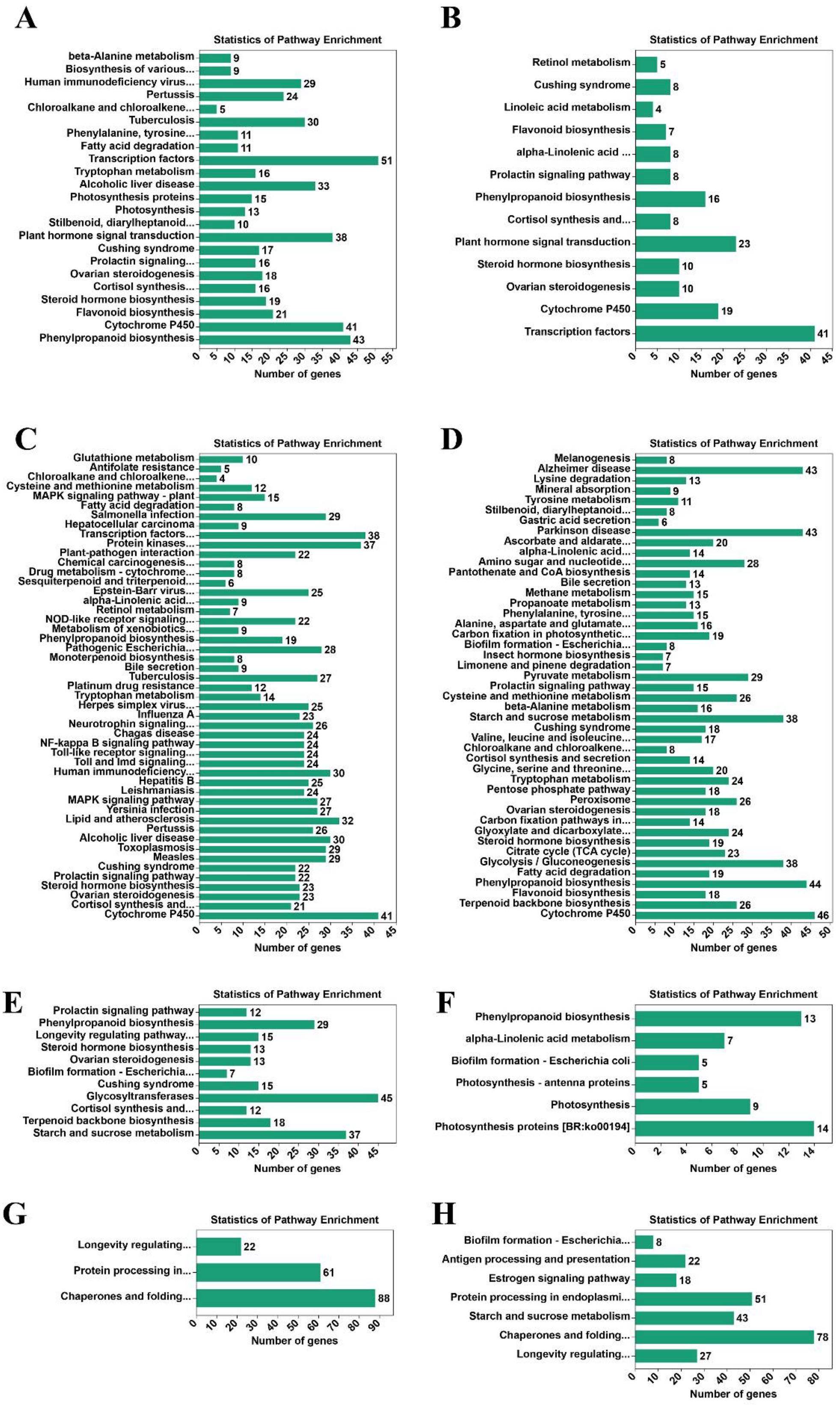

Figure S4. Significantly enriched KEGG pathways. The x-axis represents the number of genes, and the y-axis represents the KEGG pathway. Bar plot of KEGG analysis for the DEGs identified in TA1 vs. UA1 (A), TA2 vs. UA2 (B), TA3 vs. UA3 (C), TA2 vs. TA1 (D), TA3 vs. TA1 (E), TA3 vs. TA2 (F), UA2 vs. UA1 (G), and UA3 vs. UA1 (H), respectively.

Table S1. Primer pairs used in qRT-PCR assays in this study

Gene id	Gene name	Primer pairs
-	*Ubiquitin*	F: CCAGATCAATCTTGATTCCACC<br>R: ACCTAAACCAGAGCCCCCTA
-	*Histone*	F: GTACCGCTACCGGAGGGAAGTTGAAGA<br>R: CTTCTTGGGCGACTTGGTAGCCTTGGT
evm.model.Scaffold306.41	*MYB2*	F: GCAACCACCAATAGCCCTGT<br>R: TCACCACTCCAATTCCAAACA
evm.model.Scaffold6.47	*BHLH25*	F: TCCTTGTGATTCCTTCATTTCG<br>R: TGAGCCTCTCTCGTCTCTTCCT
evm.model.Scaffold10.269	*WRKY75*	F: TGATGGCTATCGTTGGAGGA<br>R: CATTTGGCTGAGGATGTGCT
evm.model.Scaffold38.68	*SSL6*	F: ATCCCAAGACCAAGAGCACC<br>R: GAATATTATCCGGCAAGCCAG
evm.model.Scaffold33.243	*SYP121*	F: AGCGAGATTCAGGAGAGGCA<br>R: CGACCAAGATGATGATGAGGAG
evm.model.Scaffold22.170	*PHT1-4*	F: AGAGGCGGAGGAAGAGAAAGT<br>R: TGAGGAGAGCGATGAGGGTT
evm.model.Scaffold31.97	*CYP749A20*	F: TTATATCCCCCTGTTTTTGTCG<br>R: TGTTCTTCGTTGCTTTCGCT
evm.model.Scaffold42.11	*SRG1*	F: AGCCGGTCATTAGGGTCCAA<br>R: CATCATCTCCTCCCGAAGTTTT
evm.model.Scaffold11.100	*C4*	F: GAGTCCAATACGAGCCCCAA<br>R: CGTCGCTTCAATTTCCCACC
evm.model.Scaffold10.221	*LHT1*	F: TACATTCCCTTTCTTCGGTGG<br>R: GCTTGGATGATGATTTGCCTC

Table S2. Differential expression of genes corresponding to transcription factors (TFs)

Gene id	TF family	TA1 vs. UA1	TA2 vs. UA2	TA3 vs. UA3
evm.model.Scaffold140.13	Aldo_ket_red	-	-	Up
evm.model.Scaffold465.33	AP2	-	-	-
novel.6374	AP2	Down	-	-
evm.model.Scaffold392.15	AP2	-	-	-
evm.model.Scaffold18.107	AP2	-	-	-
evm.model.Scaffold117.64	AP2	-	-	-
evm.model.Scaffold267.16	AP2	-	Up	-
evm.model.Scaffold6.337	AP2	-	Up	-
evm.model.Scaffold27.161	AP2	-	Up	-
evm.model.Scaffold321.39	AP2	-	-	-
evm.model.Scaffold32.132	AP2	-	Up	Up
evm.model.Scaffold9.43	AP2	-	Up	-
evm.model.Scaffold35.9	AP2	-	-	-
evm.model.Scaffold25.196	AP2	-	Up	-
evm.model.Scaffold9.119	AP2	-	Up	Up
evm.model.Scaffold29.48	AP2	-	-	-
evm.model.Scaffold55.27	AP2	-	-	-
evm.model.Scaffold74.54	AP2	Up	Up	Up
evm.model.Scaffold13.71	AP2	Up	Up	Up
evm.model.Scaffold13.30	AP2	-	-	Up
evm.model.Scaffold24.108	AP2	-	Up	Up
evm.model.Scaffold95.49	AP2	-	Up	-
evm.model.Scaffold267.44	AP2	-	Up	-
evm.model.Scaffold95.51	AP2	-	Up	-
evm.model.Scaffold45.189	AP2	-	-	-
evm.model.Scaffold1.242	AP2	-	-	-
evm.model.Scaffold307.28	AP2	Up	Up	Up
evm.model.Scaffold287.18	AP2	-	Up	Up
evm.model.Scaffold477.30	AP2	-	Up	-
evm.model.Scaffold18.313	AP2	-	-	-
evm.model.Scaffold46.95	AP2	-	Up	-
evm.model.Scaffold24.107	AP2	-	Up	Up
evm.model.Scaffold21.231	AP2	-	Up	-
evm.model.Scaffold13.31	AP2	-	Up	Up
evm.model.Scaffold8.30	AP2	-	-	Up
evm.model.Scaffold147.8	AP2	-	-	Up
evm.model.Scaffold22.163	AP2	-	-	-
evm.model.Scaffold2.274	AP2	-	-	-
evm.model.Scaffold11.262	AP2	-	-	-
evm.model.Scaffold36.141	AP2	-	-	-
evm.model.Scaffold141.1	AP2	-	-	-
evm.model.Scaffold5.465	AP2	-	Up	Up

evm.model.Scaffold43.63	AP2	-	Up	Up
evm.model.Scaffold137.11	AP2	Down	-	-
evm.model.Scaffold70.42	AP2	-	-	-
evm.model.Scaffold26.158	AP2	-	-	-
evm.model.Scaffold112.45	AP2	Up	-	Up
evm.model.Scaffold53.50	AP2	Up	Up	Up
evm.model.Scaffold9.78	AP2	Down	Up	-
evm.model.Scaffold1.344	AP2	-	-	-
evm.model.Scaffold22.161	AP2	-	-	Up
evm.model.Scaffold131.31	AP2	Down	-	-
evm.model.Scaffold220.37	AP2	-	Up	Up
evm.model.Scaffold32.111	AP2	-	-	-
evm.model.Scaffold26.89	AP2	-	Up	-
evm.model.Scaffold32.108	AP2	-	Up	-
evm.model.Scaffold1.505	AP2	Down	-	-
evm.model.Scaffold7.339	AP2	-	-	-
evm.model.Scaffold38.135	AP2	Up	Up	Up
evm.model.Scaffold18.108	AP2	-	-	-
evm.model.Scaffold117.65	AP2	-	-	-
evm.model.Scaffold31.196	AP2	-	Up	-
evm.model.Scaffold10.324	AP2	-	-	-
evm.model.Scaffold27.23	AP2	-	-	-
evm.model.Scaffold135.35	ARF	Up	-	Up
evm.model.Scaffold322.5	ARF	-	-	-
evm.model.Scaffold27.200	ARF	-	-	-
evm.model.Scaffold131.63	ARF	-	Up	Up
evm.model.Scaffold7.200	B3	Up	-	-
evm.model.Scaffold49.32	B3	-	-	-
evm.model.Scaffold72.4	B3	Up	Up	-
evm.model.Scaffold62.139	B3	-	-	-
evm.model.Scaffold3.630	B3	-	-	Up
evm.model.Scaffold206.97	B3	-	-	-
evm.model.Scaffold6.294	B3	Down	-	-
evm.model.Scaffold6.293	B3	-	-	-
evm.model.Scaffold62.118	B3	-	-	-
evm.model.Scaffold6.326	B3	-	-	-
evm.model.Scaffold6.325	B3	-	-	-
evm.model.Scaffold6.291	B3	Up	-	-
evm.model.Scaffold6.288	B3	-	-	Up
evm.model.Scaffold3.487	B3	-	-	-
evm.model.Scaffold6.267	B3	-	-	-
evm.model.Scaffold9.422	B3	-	-	-
evm.model.Scaffold171.24	B3	Up	-	Up
evm.model.Scaffold65.17	B3	-	-	-

evm.model.Scaffold23.288	B3	-	Up	-
evm.model.Scaffold1.414	Bromodomain	-	-	-
evm.model.Scaffold222.58	Bromodomain	-	-	-
evm.model.Scaffold178.27	bZIP_1	-	-	Up
evm.model.Scaffold18.16	bZIP_1	-	-	-
evm.model.Scaffold6.343	bZIP_1	Down	-	-
evm.model.Scaffold267.4	bZIP_1	-	Down	-
evm.model.Scaffold27.171	bZIP_1	-	Down	-
evm.model.Scaffold7.303	bZIP_1	Up	-	-
evm.model.Scaffold255.4	bZIP_1	-	Up	-
evm.model.Scaffold6.232	bZIP_1	-	-	-
evm.model.Scaffold27.79	bZIP_1	-	-	Down
evm.model.Scaffold135.38	bZIP_1	-	Up	Up
evm.model.Scaffold257.55	bZIP_1	Down	-	-
evm.model.Scaffold2.100	bZIP_1	Up	-	-
evm.model.Scaffold178.3	bZIP_1	-	-	Up
evm.model.Scaffold1.342	bZIP_1	-	-	Up
evm.model.Scaffold2.353	bZIP_1	-	-	-
evm.model.Scaffold68.86	bZIP_1	Up	-	-
evm.model.Scaffold4.222	bZIP_1	Up	-	-
evm.model.Scaffold64.58	bZIP_1	Up	-	Up
evm.model.Scaffold31.203	bZIP_1	Down	-	-
evm.model.Scaffold192.66	bZIP_1	Up	Up	Up
evm.model.Scaffold23.253	bZIP_1	-	-	-
evm.model.Scaffold23.68	bZIP_1	-	-	-
evm.model.Scaffold206.21	bZIP_1	-	-	-
evm.model.Scaffold28.63	bZIP_2	-	-	-
evm.model.Scaffold15.103	bZIP_2	-	-	-
evm.model.Scaffold101.149	CBFB_NFYA	Up	-	-
evm.model.Scaffold12.114	CBFB_NFYA	-	-	-
evm.model.Scaffold160.49	CBFD_NFYB_HMF	Up	-	-
evm.model.Scaffold160.50	CBFD_NFYB_HMF	Up	-	-
evm.model.Scaffold1.547	CBFD_NFYB_HMF	-	Up	Up
evm.model.Scaffold44.123	CBFD_NFYB_HMF	-	-	-
evm.model.Scaffold8.230	CBFD_NFYB_HMF	-	-	-
evm.model.Scaffold1.775	CBFD_NFYB_HMF	-	-	-
evm.model.Scaffold19.265	CBFD_NFYB_HMF	Down	-	-
evm.model.Scaffold26.45	DOG1	Up	-	-
evm.model.Scaffold42.8	DOG1	-	-	Up
evm.model.Scaffold548.13	DUF296	Down	-	-
evm.model.Scaffold4.193	DUF296	-	-	Up
evm.model.Scaffold565.17	E3F_TDP	-	-	-
evm.model.Scaffold313.8	FAR1	-	-	-
evm.model.Scaffold15.39	FYVE	-	-	-

evm.model.Scaffold92.66	FYVE	-	-	-
evm.model.Scaffold22.181	FYVE	-	-	-
evm.model.Scaffold2.476	GATA	-	-	Down
evm.model.Scaffold32.92	GATA	-	-	-
evm.model.Scaffold4.267	GATA	-	-	-
evm.model.Scaffold16.271	GATA	-	Up	-
evm.model.Scaffold62.39	GATA	Down	-	-
evm.model.Scaffold135.24	GATA	Down	-	-
evm.model.Scaffold7.323	GATA	-	-	-
evm.model.Scaffold131.48	GATA	-	-	-
evm.model.Scaffold641.2	GATA	-	-	-
evm.model.Scaffold68.139	GATA	-	-	-
evm.model.Scaffold41.37	GRAS	-	-	-
evm.model.Scaffold5.267	GRAS	-	-	-
evm.model.Scaffold221.15	GRAS	-	Up	-
evm.model.Scaffold33.25	GRAS	-	Up	-
evm.model.Scaffold52.21	GRAS	Down	Up	-
evm.model.Scaffold52.22	GRAS	-	Up	Up
evm.model.Scaffold20.291	GRAS	-	-	Up
evm.model.Scaffold44.37	GRAS	-	-	-
evm.model.Scaffold19.245	GRAS	-	Up	-
evm.model.Scaffold54.85	GRAS	-	-	-
evm.model.Scaffold13.211	GRAS	-	-	-
evm.model.Scaffold583.2	GRAS	-	-	-
evm.model.Scaffold211.4	GRAS	-	-	-
evm.model.Scaffold1.774	GRAS	-	-	-
evm.model.Scaffold474.2	GRAS	Down	Up	-
evm.model.Scaffold11.102	GRAS	-	Up	-
evm.model.Scaffold25.189	GRAS	-	-	-
evm.model.Scaffold11.229	GRAS	-	-	-
evm.model.Scaffold87.5	GRAS	-	-	-
evm.model.Scaffold10.112	HALZ	Down	-	-
evm.model.Scaffold1.448	HALZ	-	-	-
evm.model.Scaffold11.274	HD-ZIP_N	Down	-	Down
evm.model.Scaffold61.10	Helicase_C	-	-	Up
evm.model.Scaffold61.76	HLH	Down	-	-
evm.model.Scaffold51.17	HLH	-	-	-
evm.model.Scaffold201.47	HLH	-	-	-
evm.model.Scaffold26.94	HLH	Down	-	-
evm.model.Scaffold29.149	HLH	Up	-	Up
evm.model.Scaffold34.79	HLH	-	Up	-
evm.model.Scaffold6.669	HLH	-	-	-
evm.model.Scaffold211.17	HLH	-	-	-
evm.model.Scaffold1.789	HLH	-	-	-

evm.model.Scaffold17.142	HLH	Up	-	-
evm.model.Scaffold17.169	HLH	Up	-	-
evm.model.Scaffold17.171	HLH	-	-	-
evm.model.Scaffold133.2	HLH	-	-	Up
evm.model.Scaffold401.3	HLH	-	-	-
evm.model.Scaffold1.376	HLH	Up	Up	Up
evm.model.Scaffold151.42	HLH	Up	-	Up
evm.model.Scaffold28.184	HLH	Up	-	-
evm.model.Scaffold6.47	HLH	Up	Up	Up
evm.model.Scaffold1.379	HLH	-	-	-
evm.model.Scaffold60.31	HLH	Up	-	Up
evm.model.Scaffold31.188	HLH	-	-	-
evm.model.Scaffold107.31	HLH	-	-	-
evm.model.Scaffold216.24	HLH	-	Down	-
evm.model.Scaffold29.150	HLH	Up	-	Up
evm.model.Scaffold5.546	HLH	Up	-	Up
evm.model.Scaffold43.149	HLH	-	-	Up
evm.model.Scaffold46.105	HLH	Down	-	-
evm.model.Scaffold5.443	HLH	-	-	-
evm.model.Scaffold43.38	HLH	-	-	-
evm.model.Scaffold2.401	HLH	Down	-	-
evm.model.Scaffold307.46	HLH	Down	-	-
evm.model.Scaffold13.84	HLH	Down	-	-
evm.model.Scaffold70.20	HLH	-	-	-
evm.model.Scaffold356.7	HLH	-	-	-
evm.model.Scaffold95.41	HLH	Up	-	Up
evm.model.Scaffold1.445	HLH	Down	-	-
evm.model.Scaffold2.618	HLH	-	-	-
evm.model.Scaffold14.184	HLH	-	-	-
evm.model.Scaffold13.91	HLH	-	-	-
evm.model.Scaffold235.11	HLH	-	-	-
evm.model.Scaffold8.93	HLH	Down	-	-
evm.model.Scaffold62.69	HLH	-	-	-
evm.model.Scaffold59.28	HLH	-	-	-
evm.model.Scaffold192.75	HLH	-	-	-
evm.model.Scaffold56.65	HLH	-	-	-
evm.model.Scaffold3.521	HLH	-	-	-
evm.model.Scaffold448.31	HLH	-	-	-
evm.model.Scaffold4.220	HLH	-	-	-
evm.model.Scaffold68.84	HLH	-	-	-
evm.model.Scaffold22.131	HLH	Up	-	-
evm.model.Scaffold206.52	HLH	-	Up	Up
evm.model.Scaffold3.252	HLH	-	-	Up
evm.model.Scaffold206.78	HLH	Up	-	Up

evm.model.Scaffold32.21	HLH	Down	-	-
evm.model.Scaffold9.362	HLH	-	-	Up
evm.model.Scaffold147.29	HLH	-	-	-
evm.model.Scaffold108.29	HMG_box	-	-	-
evm.model.Scaffold10.32	Homeobox	-	-	-
evm.model.Scaffold3.538	Homeobox	Up	-	-
evm.model.Scaffold36.142	HSF_DNA-bind	Up	Up	Up
evm.model.Scaffold28.4	HSF_DNA-bind	-	-	-
evm.model.Scaffold13.159	HSF_DNA-bind	-	-	Up
evm.model.Scaffold5.307	HSF_DNA-bind	-	-	-
evm.model.Scaffold497.7	HSF_DNA-bind	-	-	-
evm.model.Scaffold147.60	HSF_DNA-bind	Down	-	-
evm.model.Scaffold2.260	HSF_DNA-bind	-	-	-
evm.model.Scaffold39.111	HSF_DNA-bind	Up	-	Up
evm.model.Scaffold46.42	HSF_DNA-bind	-	-	Up
evm.model.Scaffold1.214	HSF_DNA-bind	-	-	Up
evm.model.Scaffold24.193	HSF_DNA-bind	-	-	-
evm.model.Scaffold36.9	HSF_DNA-bind	Up	Up	Up
evm.model.Scaffold141.2	HSF_DNA-bind	Up	Up	Up
evm.model.Scaffold257.48	HSP70	-	-	-
evm.model.Scaffold18.113	HSP70	-	-	Up
evm.model.Scaffold117.70	HSP70	-	-	Up
evm.model.Scaffold85.88	HSP70	-	-	Up
evm.model.Scaffold101.137	HSP70	-	-	-
evm.model.Scaffold21.69	HSP70	-	-	-
evm.model.Scaffold2.414	K-box	-	-	-
evm.model.Scaffold363.9	K-box	-	Down	-
evm.model.Scaffold159.16	K-box	-	Down	-
evm.model.Scaffold1.123	MFMR	Down	-	-
evm.model.Scaffold222.47	MYB DNA-binding	-	-	-
evm.model.Scaffold10.207	MYB DNA-binding	-	-	-
evm.model.Scaffold59.53	MYB DNA-binding	-	-	-
evm.model.Scaffold92.92	MYB DNA-binding	-	-	-
evm.model.Scaffold33.168	MYB DNA-binding	-	-	-
evm.model.Scaffold4.262	MYB DNA-binding	-	-	-
evm.model.Scaffold68.135	MYB DNA-binding	-	-	-
evm.model.Scaffold36.49	MYB DNA-binding	Down	-	-
evm.model.Scaffold141.39	MYB DNA-binding	-	-	-
evm.model.Scaffold84.45	MYB DNA-binding	-	-	-
evm.model.Scaffold98.8	MYB DNA-binding	-	-	-
evm.model.Scaffold223.9	MYB DNA-binding	Down	-	-
evm.model.Scaffold4.231	MYB DNA-binding	Up	-	-
evm.model.Scaffold106.12	MYB DNA-binding	Up	-	-
evm.model.Scaffold18.14	MYB DNA-binding	Down	-	-

evm.model.Scaffold68.98	MYB DNA-binding	Up	-	-
evm.model.Scaffold42.9	MYB DNA-binding	Down	Up	-
evm.model.Scaffold610.1	MYB DNA-binding	-	-	-
evm.model.Scaffold68.99	MYB DNA-binding	-	-	-
evm.model.Scaffold54.139	MYB DNA-binding	Down	-	-
evm.model.Scaffold263.8	MYB DNA-binding	-	-	-
evm.model.Scaffold1.45	MYB DNA-binding	-	-	-
evm.model.Scaffold2.212	MYB DNA-binding	-	Up	-
evm.model.Scaffold42.203	MYB DNA-binding	-	-	-
evm.model.Scaffold42.162	MYB DNA-binding	-	-	-
evm.model.Scaffold1.656	MYB DNA-binding	-	-	-
evm.model.Scaffold224.56	MYB DNA-binding	-	-	-
evm.model.Scaffold62.114	MYB DNA-binding	-	-	-
evm.model.Scaffold142.11	MYB DNA-binding	-	-	Up
evm.model.Scaffold5.237	MYB DNA-binding	Up	Up	-
evm.model.Scaffold85.104	MYB DNA-binding	-	Up	-
evm.model.Scaffold9.369	MYB DNA-binding	-	-	-
evm.model.Scaffold154.10	MYB DNA-binding	-	-	-
evm.model.Scaffold403.23	MYB DNA-binding	-	Up	-
evm.model.Scaffold671.2	MYB DNA-binding	-	Up	-
evm.model.Scaffold25.45	MYB DNA-binding	-	Down	-
evm.model.Scaffold18.359	MYB DNA-binding	-	-	-
evm.model.Scaffold494.8	MYB DNA-binding	Up	-	-
evm.model.Scaffold306.41	MYB DNA-binding	-	Up	Up
evm.model.Scaffold400.9	MYB DNA-binding	-	Up	Up
evm.model.Scaffold322.28	MYB DNA-binding	-	-	Up
evm.model.Scaffold7.278	MYB DNA-binding	-	-	Up
evm.model.Scaffold2.370	MYB DNA-binding	Up	-	-
evm.model.Scaffold5.507	MYB DNA-binding	-	-	-
evm.model.Scaffold43.111	MYB DNA-binding	-	-	-
evm.model.Scaffold9.87	MYB DNA-binding	-	-	-
evm.model.Scaffold26.208	MYB DNA-binding	-	Up	-
evm.model.Scaffold1.257	MYB DNA-binding	-	Up	-
evm.model.Scaffold16.20	MYB DNA-binding	-	-	-
evm.model.Scaffold2.292	MYB DNA-binding	-	-	-
evm.model.Scaffold30.119	MYB DNA-binding	Up	-	Up
evm.model.Scaffold20.252	MYB DNA-binding	-	-	Down
evm.model.Scaffold85.28	MYB DNA-binding	-	-	-
evm.model.Scaffold6.610	MYB DNA-binding	-	-	-
evm.model.Scaffold44.127	MYB DNA-binding	-	-	-
evm.model.Scaffold4.447	MYB DNA-binding	-	-	Down
evm.model.Scaffold29.182	MYB DNA-binding	Up	Up	Up
evm.model.Scaffold1.477	MYB DNA-binding	Up	-	-
evm.model.Scaffold270.15	MYB DNA-binding	Up	Up	Up

evm.model.Scaffold70.51	MYB DNA-binding	-	-	-
evm.model.Scaffold8.32	MYB DNA-binding	Up	-	Up
evm.model.Scaffold6.686	MYB DNA-binding	-	Up	-
evm.model.Scaffold147.2	MYB DNA-binding	-	Up	Up
evm.model.Scaffold22.175	MYB DNA-binding	-	Up	Up
evm.model.Scaffold5.334	MYB DNA-binding	Up	-	Up
evm.model.Scaffold8.383	MYB DNA-binding	Up	Up	-
evm.model.Scaffold204.34	MYB DNA-binding	Up	Up	-
evm.model.Scaffold117.34	MYB DNA-binding	Up	-	-
evm.model.Scaffold18.78	MYB DNA-binding	Up	-	-
evm.model.Scaffold384.5	MYB DNA-binding	Up	-	-
evm.model.Scaffold206.60	MYB DNA-binding	Up	-	-
evm.model.Scaffold7.153	MYB DNA-binding	-	Down	-
evm.model.Scaffold3.515	MYB DNA-binding	-	-	-
evm.model.Scaffold402.8	MYB DNA-binding	-	-	-
evm.model.Scaffold6.140	MYB DNA-binding	Up	-	Up
evm.model.Scaffold28.54	MYB DNA-binding	Up	Up	-
evm.model.Scaffold26.215	MYB DNA-binding	Up	Up	-
evm.model.Scaffold167.17	MYB DNA-binding	Up	Up	-
evm.model.Scaffold5.397	MYB DNA-binding	-	-	-
evm.model.Scaffold131.54	MYB DNA-binding	-	Up	-
evm.model.Scaffold321.14	MYB DNA-binding	-	-	-
evm.model.Scaffold32.48	MYB DNA-binding	-	-	-
evm.model.Scaffold67.8	MYB DNA-binding	-	-	-
evm.model.Scaffold28.108	NAM	-	Up	-
evm.model.Scaffold6.89	NAM	-	Up	-
evm.model.Scaffold1.720	NAM	-	-	-
evm.model.Scaffold20.107	NAM	-	-	-
evm.model.Scaffold1.802	NAM	Up	-	Up
evm.model.Scaffold85.112	NAM	Up	Up	Up
evm.model.Scaffold8.386	NAM	Up	Up	Up
evm.model.Scaffold23.174	NAM	-	-	-
evm.model.Scaffold1.732	NAM	-	-	Down
evm.model.Scaffold7.204	NAM	Up	-	-
evm.model.Scaffold175.42	NAM	-	Up	-
evm.model.Scaffold68.4	NAM	Down	-	-
evm.model.Scaffold17.99	NAM	Down	-	-
evm.model.Scaffold10.290	NAM	Up	-	Up
evm.model.Scaffold26.135	NAM	Up	-	-
evm.model.Scaffold155.20	NAM	Up	-	Up
evm.model.Scaffold32.31	NAM	Up	-	-
evm.model.Scaffold203.47	NAM	-	-	Down
evm.model.Scaffold83.48	NAM	-	Up	Up
evm.model.Scaffold1.665	NAM	Down	-	-

evm.model.Scaffold3.643	NAM	-	-	Down
evm.model.Scaffold8.41	NAM	-	-	Down
evm.model.Scaffold455.33	NAM	-	-	-
evm.model.Scaffold1.693	NAM	-	Up	Up
evm.model.Scaffold25.232	NAM	-	Up	Up
evm.model.Scaffold5.599	NAM	-	-	-
evm.model.Scaffold3.37	NAM	Up	-	-
evm.model.Scaffold38.154	NAM	-	-	-
evm.model.Scaffold3.628	NAM	-	-	-
evm.model.Scaffold14.181	NAM	-	-	-
evm.model.Scaffold289.41	NAM	-	-	-
evm.model.Scaffold19.162	NAM	Up	-	-
evm.model.Scaffold23.363	NAM	-	-	-
evm.model.Scaffold61.34	NAM	-	-	-
evm.model.Scaffold547.1	NAM	-	-	Up
evm.model.Scaffold5.312	PLATZ	-	-	-
evm.model.Scaffold23.154	PLATZ	-	-	-
evm.model.Scaffold26.120	SAP	-	-	-
evm.model.Scaffold94.19	SAP	-	-	-
evm.model.Scaffold306.21	SBP	-	-	-
evm.model.Scaffold39.144	SBP	-	-	Down
evm.model.Scaffold59.47	SBP	-	Up	-
evm.model.Scaffold33.163	SBP	-	-	-
evm.model.Scaffold9.170	SBP	-	-	-
evm.model.Scaffold24.44	SNF2_N	-	-	Up
evm.model.Scaffold61.9	SNF2_N	-	-	Up
evm.model.Scaffold6.521	SRF-TF	-	-	-
evm.model.Scaffold137.85	SRF-TF	-	-	-
evm.model.Scaffold224.63	SRF-TF	Up	-	Up
evm.model.Scaffold28.198	SRF-TF	-	-	-
evm.model.Scaffold2.726	SRF-TF	-	-	-
evm.model.Scaffold1.422	SRF-TF	-	-	-
evm.model.Scaffold6.485	SRF-TF	-	Down	-
evm.model.Scaffold60.57	SRF-TF	Up	-	-
evm.model.Scaffold182.41	SRF-TF	-	Up	-
evm.model.Scaffold346.7	SRF-TF	-	-	-
evm.model.Scaffold70.10	SRF-TF	-	-	-
evm.model.Scaffold18.298	SRF-TF	-	Down	-
evm.model.Scaffold8.430	SRF-TF	-	-	-
evm.model.Scaffold3.331	SRF-TF	-	-	-
evm.model.Scaffold11.158	SRF-TF	-	-	-
evm.model.Scaffold2.469	START	-	-	-
evm.model.Scaffold55.37	START	-	-	-
evm.model.Scaffold49.84	START	-	-	-

evm.model.Scaffold257.25	START	-	-	-
evm.model.Scaffold47.6	Tau96	-	-	-
evm.model.Scaffold155.81	TCP	Up	-	Up
evm.model.Scaffold298.42	TCP	-	-	-
evm.model.Scaffold95.29	TCP	-	-	-
evm.model.Scaffold10.275	TCP	-	-	-
evm.model.Scaffold27.48	TCP	Down	-	-
evm.model.Scaffold6.621	TCP	-	-	Down
evm.model.Scaffold92.64	TCP	-	-	Down
evm.model.Scaffold58.22	TCP	Down	-	-
evm.model.Scaffold262.2	TCP	-	-	-
evm.model.Scaffold36.14	TFIIB,TF_Zn_Ribbon	-	-	-
evm.model.Scaffold101.127	TPR_16,TPR_19,TPR_6,TPR_7,TPR_9	-	-	-
evm.model.Scaffold1.247	WRKY	-	-	Up
evm.model.Scaffold229.22	WRKY	-	-	-
evm.model.Scaffold4.210	WRKY	-	-	-
evm.model.Scaffold68.74	WRKY	-	-	-
evm.model.Scaffold306.54	WRKY	-	Up	-
evm.model.Scaffold7.243	WRKY	Up	-	Up
evm.model.Scaffold155.37	WRKY	-	-	-
evm.model.Scaffold3.221	WRKY	Up	-	Up
evm.model.Scaffold3.179	WRKY	-	Up	Up
evm.model.Scaffold7.128	WRKY	-	Up	Up
evm.model.Scaffold21.244	WRKY	-	Up	Up
evm.model.Scaffold117.83	WRKY	-	-	Up
evm.model.Scaffold18.126	WRKY	-	-	Up
evm.model.Scaffold9.430	WRKY	-	Up	Up
evm.model.Scaffold10.409	WRKY	-	Up	-
evm.model.Scaffold10.36	WRKY	Up	-	Up
evm.model.Scaffold271.5	WRKY	Up	-	-
evm.model.Scaffold129.9	WRKY	-	-	Up
evm.model.Scaffold314.23	WRKY	-	-	Up
evm.model.Scaffold9.167	WRKY	Up	Up	Up
evm.model.Scaffold10.269	WRKY	-	Up	Up
evm.model.Scaffold13.112	WRKY	-	-	-
evm.model.Scaffold131.93	WRKY	Up	Up	Up
evm.model.Scaffold135.59	WRKY	Up	Up	Up
evm.model.Scaffold30.90	WRKY	-	-	-
evm.model.Scaffold6.65	WRKY	-	-	Up
evm.model.Scaffold31.48	WRKY	-	-	Up
evm.model.Scaffold49.21	WRKY	Up	-	Up
evm.model.Scaffold19.164	WRKY	Up	Up	Up
evm.model.Scaffold457.11	WRKY	Down	-	-

evm.model.Scaffold306.65	WRKY	Down	Down	-
evm.model.Scaffold7.293	WRKY	-	-	-
evm.model.Scaffold8.405	WRKY	Up	Up	Up
evm.model.Scaffold111.13	WRKY	-	-	-
evm.model.Scaffold19.311	WRKY	-	Up	Up
evm.model.Scaffold171.22	WRKY	Up	Up	Up
evm.model.Scaffold369.3	WRKY	-	Up	Up
evm.model.Scaffold7.283	WRKY	Up	-	-
evm.model.Scaffold7.87	WRKY	Down	-	-
evm.model.Scaffold31.217	WRKY	-	-	Up
evm.model.Scaffold33.50	WRKY	-	Up	Up
evm.model.Scaffold70.29	WRKY	-	-	Up
evm.model.Scaffold13.356	zf-Dof	-	-	-
evm.model.Scaffold92.47	ZF-HD_dimer	Down	-	-
